# Comparative genome analysis of cluster EF bacteriophages Ajin and OverHedge isolated from soil in Tennessee

**DOI:** 10.1128/mra.00925-24

**Published:** 2024-09-30

**Authors:** Sergei A. Markov, Paul Y. Atuahene, Clayton W. Barnes, Taquerra Butler, Stephan D. Cooper, Emma M. Covel, Kayla R. Diaz, Dalon Gadson, Anna C. Holt, Maegan A. Litchfield, Alexis J. Nefe, Comfort E. Ogbu, Kiara M. Rupp, Falisaty Simpson, Elyse Wood

**Affiliations:** 1 Department of Biology, Austin Peay State University, Clarksville, Tennessee, USA; Department of Biology, Queens College, Queens, New York, USA

**Keywords:** bacteriophages, genome analysis

## Abstract

Bacteriophages Ajin and OverHedge were isolated from soil in Tennessee using the bacterium *Microbacterium foliorum*. Ajin and OverHedge (cluster EF) have a genome of 56,993 bp and 56,559 bp, containing 86 and 81 predicted genes, respectively. The Ajin genome has unique genes, phosphatase and glycosyltransferase, compared to the OverHedge.

## ANNOUNCEMENT

To expand our knowledge of actinobacteriophages, bacteriophages Ajin and OverHedge were found and characterized using *Microbacterium foliorum* NRRL B-24224 (1, 2).

Ajin and OverHedge were isolated from soil (temperature was 11°C and 18°C, respectively) in Tennessee (GPS coordinates: 36.475315 N, 87.244529 W and 36.534222 N, 87.352528 W, correspondingly) using standard procedures described in https://seaphagesphagediscoveryguide.helpdocsonline.com/home. Soil samples underwent incubation in peptone yeast calcium (PYCa) medium for 2 hours. The resulting suspension was filtered through a 0.22-mm pore filter, and the filtrate was inoculated with *M. foliorum* while shaking at 250 rpm for 2 days at 30°C. Bacteriophages were purified through three rounds of plating. The phages formed clear small plaques ranging from 0.5 to 1.0 mm in diameter (see plaque photos at https://phagesdb.org/institutions/APSU/).

**Fig 1:**
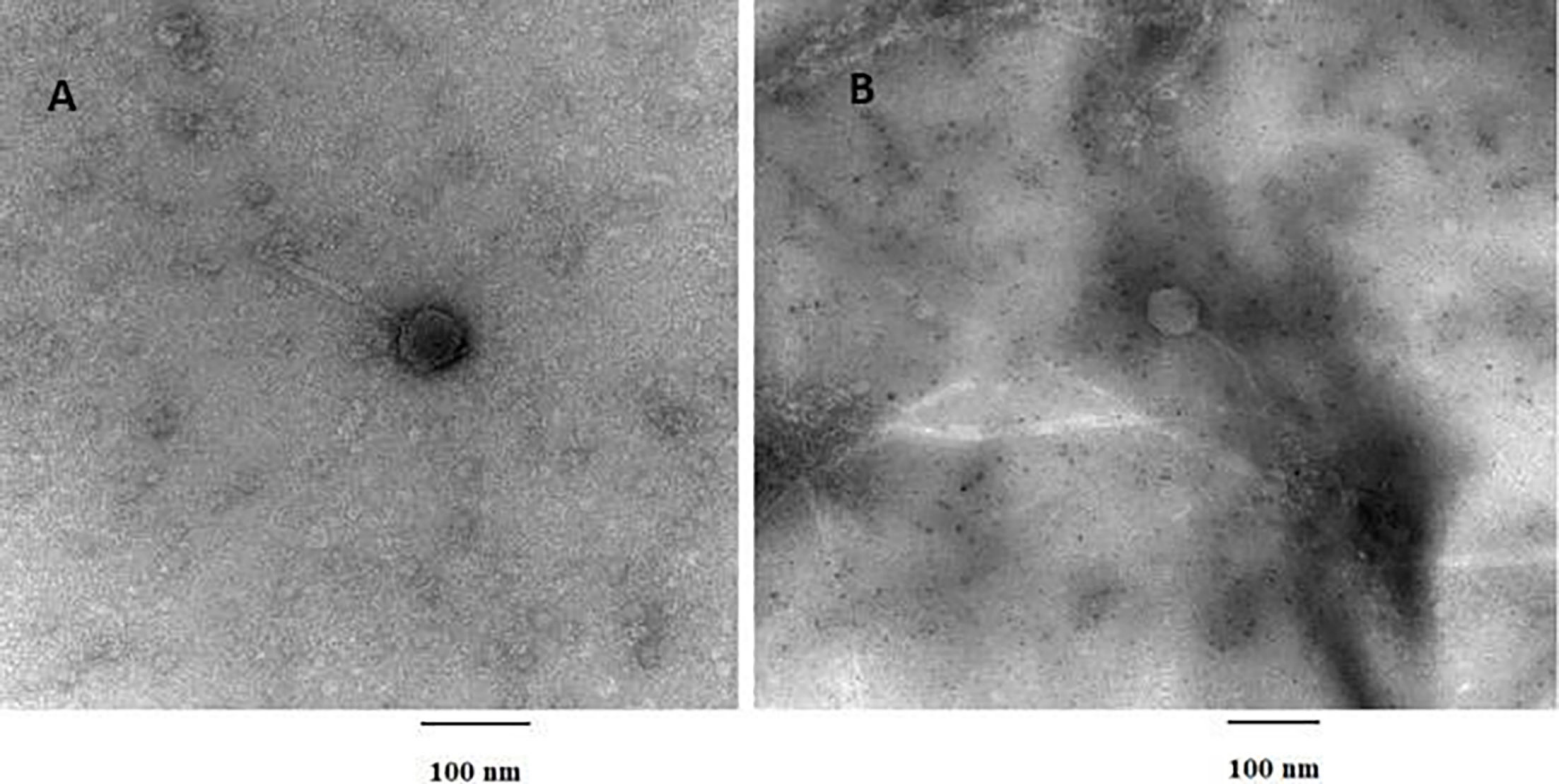
Transmission electron microscopy photos of bacteriophages Ajin (**A**) and OverHedge (**B**) showing a siphovirus morphology with an icosahedral capsid of diameter 52–54 nm and a 132–134 nm long flexible tail (*n* = 7). Bacteriophage samples were stained using 1% uranyl acetate on grids attached to Pelco Tabs (Ted Peller, Inc., Redding, CA). The Hitachi H-7650 Transmission Electron Microscope (Tokyo, Japan) was used with an accelerating voltage of 100 kV.

Bacteriophages morphologies were determined by transmission electron microscopy with negative staining revealing a siphovirus morphology with an icosahedral capsid and flexible tail (Fig. 1). Both phages are under the following taxonomy ranks: Realm *Duplodnaviria*; Kingdom *Heunggongvirae*; Phylum *Uroviricota*; Class *Caudoviricetes*.

Bacteriophage DNAs were isolated using the Wizard DNA Clean-Up Kit (Promega, Madison, WI) and prepared for sequencing using the Ultra II Library Kit (NEB, Ipswich, MA). For library preparations, 300 ng of DNA was used for both phages. The DNA was sheared (enzymatically) as part of the library preparation protocol. It was quantified by the Qubit Broad Range DNA assay. Four cycles of PCR were used to amplify DNA during the Ultra II NEB protocol. A MiSeq instrument (v3 reagents) from Illumina (San Diego, CA) was used for DNA sequencing to yield 317,718 150-base single-end reads with 836-fold coverage for Ajin and 374,401 150-base single-end reads with 992-fold coverage for OverHedge. The raw reads with no additional QC/trimming were assembled using Newbler v2.9 and checked for genomic termini and completeness using Consed v29, as described by Russell (3).

Ajin possesses a genome of 56,993 bp with a 62.6% GC content, while OverHedge has a smaller genome (56,559 bp) with 63.8% GC content. Based on the gene content similarity (GCS) of at least 35% to bacteriophages in the Actinobacteriophage database (https://phagesdb.org/genecontent/), both bacteriophages were placed into the cluster EF (4, 5). Ajin is closely related (93.74%) to the bacteriophage TinyMiny (accession number MZ681513). OverHedge is closely related to Gilda (accession number MT818420) at 96.34%.

Bacteriophage genome annotation was conducted using DNA Master v5.23.6 (http://cobamide2.bio.pitt.edu/), Glimmer v3.02 (6), GeneMark v2.5p (7), PECAAN v20211202 (http://blog.kbrinsgd.org/), NCBI Blast (8), HHPred v3.2 (9) using the PDB_mmCIF70, Pfam v.37, NCBI Conserved Domains databases v3.19, and DeepTMHMM v1.0.24 (10). Default parameters were used, except where otherwise noted. Genome annotation revealed that Ajin has 86 predicted protein-coding genes (assigned functions for 29 genes). Bacteriophage OverHedge has 81 predicted protein-coding genes (assigned functions for 34).

The GCS revealed 53.89% gene similarity between Ajin and OverHedge, suggesting notable differences. Ajin has unique genes with known functions, phosphatase and glycosyltransferase, compared to OverHedge. Conversely, we identified three additional membrane protein genes in OverHedge. Given these discrepancies, it is plausible to classify Ajin and OverHedge into different subclusters within the EF cluster.

## Data Availability

GenBank accession numbers for Ajin and OverHedge are PP978814 and PP978795, respectively. SRA accession numbers for Ajin and OverHedge are SRX24123886 and SRX24123892, respectively.
